# Snuff Taking

**Published:** 1881-09

**Authors:** 


### SNUFF TAKING.

*General Sullivan*, of the Revolutionary army, carried his snuff loose in his vest pocket. "At times," says the *Medical World*, "he had violent pains in the head; the intervals grew shorter, and the returns more distressing, ending in *palsy*, which rendered him helpless and miserable, and put him in his grave before he was fifty years old. *The earlier in life, and the earlier in the day tobacco is used, the more pernicious is its effect on the constitution.*

